# Microbial fuel cells directly powering a microcomputer

**DOI:** 10.1016/j.jpowsour.2019.227328

**Published:** 2020-01-15

**Authors:** Xavier Alexis Walter, John Greenman, Ioannis A. Ieropoulos

**Affiliations:** Bristol BioEnergy Centre, Bristol Robotics Laboratory, T-Block, Frenchay Campus, University of the West of England (UWE), Bristol, BS16 1QY, United Kingdom

**Keywords:** Membraneless microbial fuel cell, Urine, Energy source, Practical applications, Direct power

## Abstract

Many studies have demonstrated that microbial fuel cells (MFC) can be energy-positive systems and power various low power applications. However, to be employed as a low-level power source, MFC systems rely on energy management circuitry, used to increase voltage levels and act as energy buffers, thus delivering stable power outputs. But stability comes at a cost, one that needs to be kept minimal for the technology to be deployed into society. The present study reports, for the first time, the use of a MFC system that directly and continuously powered a small application without any electronic intermediary. A cascade comprising four membrane-less MFCs modules and producing an average of 62  mA at 2550 mV (158 mW) was used to directly power a microcomputer and its screen (Gameboy Color, Nintendo®). The polarisation experiment showed that the cascade produced 164 mA, at the minimum voltage required to run the microcomputer (ca. 1.850 V). As the microcomputer only needed ≈70 mA, the cascade ran at a higher voltage (2.550 V), thus, maintaining the individual modules at a high potential (>0.55 V). Running the system at these high potentials helped avoid cell reversal, thus delivering a stable level of energy without the support of any electronics.

## Highlights

• First use of microbial fuel cells as the direct power source of a microcomputer.
• No power management or other electronic circuitry were employed.
• Continuously powered a *Game Boy Color* for 150 h.
• The system was shown to be stable for at least 120 h.

## 1 Introduction

While research on MFC was first reported in 1911 [1], bioelectrochemical systems (BES) converting the chemical energy contained in reduced organic matter into electrical energy have recently received great attention, as illustrated by the number of publications on the subject [2]. Research in BES has become a scientific field in its own right. Research on the topic has focussed on microbial fuel cells (MFC) both as a remediation technology for wastewater treatment and as a low-level renewable energy source for applications. This is because the MFC technology is the only BES that can directly convert wastewater into electrical energy [[3], [4], [5]], thus saving energy for a process that normally consumes a lot of electrical power. As an example, 0.33–1.0% of the electricity produced in the UK is consumed just for treating sewage water (2532 GWh in 2005/06) [6]. A major step in wastewater treatment is the activated sludge process, used for oxidising organic matter and removing nutrients (e.g. nitrogen, phosphorous). However, this process consumes 55.6% of the total energy required to treat wastewater [7]. Although both aspects of the technology are of importance and inherently linked, extracting energy and treating waste, the present study focuses on the power generation and its use as a stable power source for an off-the-shelf application.

At single MFC level, the maximum power transfer point is usually occurring at a voltage between 300 mV and 500 mV, depending on the design and the material employed. In addition, due to diffusion limitations and increased internal resistance, smaller MFCs are more power dense than larger ones [8]. These two factors, which are inherent to the technology, have driven the development of MFC-systems aimed at powering real-world applications that need high power densities. The common strategy pursued by researchers is to assemble a plurality of MFCs into stacks [9]. This strategy allows for the units to be electrically connected either in series or in parallel consequently increasing either the voltage or the current produced by the stack [[10], [11], [12]]. However, in the case of serial connection voltage reversal can be observed between MFCs [11,13], although this can be avoided if uniformity between MFC units is maintained. Therefore, the most common approach to reach high voltages (e.g. >1.5 V) is to electrically connect all MFCs in parallel and employ electronic circuitry to boost the voltage. Several studies have shown that MFC systems can power various applications, without voltage reversal, through the use of either DC/DC converter [[14], [15], [16], [17], [18], [19]] or capacitor banks combined with purpose-built management circuitry [4,20,21]. However, as pointed out by Chen et al. [12], the cost of MFC systems has to be kept minimal for technology to be deployed in society, and power management systems represent an additional cost that could limit the technology's implementation. Therefore, the objective here was to investigate if MFCs could directly power an off-the-shelf application without the need for any electronic circuitry.

## 2 Materials and methods

### 2.1 Reactor construction and operation

**Fig. 1:**
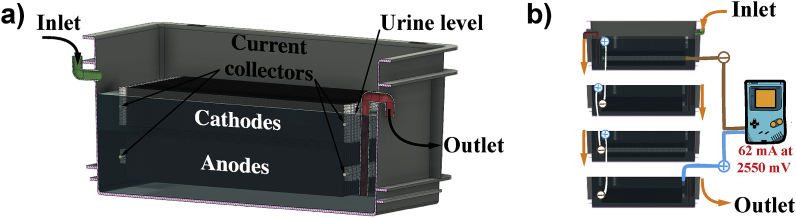
3D view of a single S-MFC module (**a**), and section view of the cascade of 4 modules and its electrical connections to the *Game Boy Color* that it powers (**b**). In (**b**) the hydraulic connections are represented in orange and the electrical connections in white.

The challenge of running large systems in the laboratory is the availability of fuel. Hence, the hypothesis was to employ relatively large size MFC modules, with respect to the target application, and operating the system under suboptimal feeding conditions, to simulate real or field environments and test the system capability in terms of voltage reversal. Following our past experience in powering application [17,18], the stack assembled here employed self-stratifying membraneless MFCs modules (S-MFCs). In such modules, a plurality of anodes and cathodes are inserted in a single container thus sharing the same electrolyte (Fig. 1a). This type of design exploits the capacity of complex microbial consortia to structure themselves and the environment in which they thrive in a succession of horizontal layers following a redox gradient: the more reduced layers are at the bottom of the electrolyte column and the more oxidised layers are at the top of the column. In S-MFC, vertical anodes are completely submerged in the reduced lower-layers and vertical cathodes are partially submerged in the more oxidised upper-layer of the system, 5 mm above the anodes, and partially exposed to air (i.e. ¾ of the height) [22,23] (Fig. 1a). The stack employed in this study comprised a single cascade consisting of 4 modules electrically connected in series (Fig. 1b). The anodes of the first module and the cathodes of the cascade's forth module served as the negative and positive terminals of the system, respectively. The assembled stack was then directly connected to the positive and negative terminals in the battery compartment of the *Game Boy Color* device (Fig. 1b). The objective was to demonstrate that MFCs could directly and continuously power an exemplar microcomputer and its screen without the use of any power management electronic circuitry.

The four S-MFC modules employed in the present report were designed following the results from previous studies investigating scalability, materials and functional parameters of urine-fed S-MFCs [[22], [23], [24]]. Each module comprised a rack of 41 cathodes placed 5 mm above a rack of 31 anodes. The electrode racks of a module were inserted in a 15L plastic Euro Stacking Containers (400 × 300 × 170mm; Plastor Ltd., UK). Each of the 2 mm thick cathodes had a projected surface area of 126 cm^2^ (42 × 300mm). The cathodes were made by hot pressing an activated-carbon (AC) and polytetrafluoroethylene (PTFE) mixture (80% AC; 20% PTFE; 280 °C) onto a stainless-steel 316 mesh acting as the current collector (8 × 8 mesh; MeshDirect, UK). For all the cathodes of each module, a total of 500 mL PTFE (60% dispersion solution) and 1 kg of activated carbon powder were employed. Each of the anodes was made from 3000 cm^2^ of 10gsm carbon veil (Technical Fibre Products Ltd, Cumbria, UK) that was folded down to a projected surface area of 150 cm^2^ (50 × 300mm). A strip of stainless-steel 316 mesh was placed in the middle as the current collector. For all the anodes of a module, a total of 9.3 m^2^ of carbon veil was used. All the electrodes of a module were connected in parallel because they were submerged in the same undiluted human urine electrolyte. The urine level was adjusted to approximatively ¾ of the cathode height, based on previous findings showing that this is the optimum running condition [23].

The stack of four S-MFC modules was assembled by stacking each module on the top of each other. As the effluent of one module was feeding into the one below, the stack consisted of a single cascade of four modules. For clarity, this system will be referred to as a cascade. To simulate the irregular feeding encountered during deployment under real condition of use [18,22], 1.8 L of urine was pumped once every 3 h, unless otherwise stated. Since each module had a displacement volume of 6.9 L (volume of urine contained by a single module), the hydraulic retention times were ≈11.5 h and 46 h for a single module and the whole cascade, respectively. Although this HRT is longer than what this design has actually been developed for (≈1.5 h HRT) [18], urine availability limited the possibility of using a shorter HRT for a long enough period to show stability. Urine was collected daily from a tank pooling together the urine donated by anonymous and supposedly healthy individuals. By the time the urine fed the cascade, it had gone through a partial hydrolysis resulting in an increased pH ranging between 8.5 and 9.3. The cascade was inoculated with a mixture consisting of 85% (v/v) of effluent from a separate and mature MFC system – also fed with urine – and 15% (v/v) of freshly collected urine.

### 2.2 Data capture and system characterisation

Once inoculated, the cascade was left in open circuit conditions overnight prior being connected to a programmable DC electronic load (BK Precision 8500, B&K Precision Corp., USA) which helps maintain a constant voltage of either 450 mV (1st phase of the experiment) or 1800 mV (2nd phase of the experiment). The current produced by the cascade was measured by monitoring the voltage drop across a wire of a known resistance (0.00371 Ω). In addition, the voltage of the cascade and of each of its modules were monitored using a data acquisition system (Agilent LXI 34972A; Farnell, UK). During the first phase of the experiment, the data were logged every 4 min, and then, every 2 min during the second phase of the experiment. The first phase of the experiment was meant to let the cascade reach steady-state under ideal conditions with all four modules connected in parallel, thus avoiding cell reversal during the inoculation phase [11,25,26]. Once a steady-state was reached under the given running conditions (HRT of 11.5 h), the second phase of the experiment started with all four modules connected in series and maintained at the constant voltage of 1800 mV (i.e. 4 times 450 mV; BK Precision 8500). Once, stability was observed, the cascade was directly connected to a *Game Boy Color* (**GBC**; Nintendo Co. Ltd., Japan).

At the end of the experimental run, once the cascade had matured, a polarisation experiment was performed by linear sweep voltammetry (LSV). Because the four S-MFC modules of the cascade were hydraulically disconnected but electrically connected in series, the polarisation experiment was carried out on the cascade as a whole. In this case, a two-electrode configuration (potentiostat Biologic SP-50) was adopted with the reference electrode channel short-circuited with the counter electrode channel. The anodes of the first-module were the negative electrode and the cathodes of the last module served as the positive electrode. The scan rate was 0.25 mV s^−1^ to avoid any overestimation [27] and scanned from OCV to 700 mV. Prior the polarisation experiments, the cascade was left under open circuit conditions (OCV) for 1 h. During the polarisation experiments, the voltages of each module were recorded independently (Agilent LXI 34972A; Farnell, UK).

## 3 Results

### 3.1 Inoculation phase: stability under potentiostatic conditions

Although having the cascade directly connected to an application from inoculation would have been of pertinence, the aim was to characterise the cascade behaviour under potentiostatic conditions prior to powering the application. Preliminary tests on the electrical energy required by the *Game Boy Color* (GBC) showed that it needed a minimum voltage of 1.8 V and 70–75 mA to run. Since the optimal operating potential for the S-MFC modules is around 450 mV [17,18], four modules electrically connected in series should have the potential to directly power the **GBC**. In order to optimise the inoculation of the cascade, all four S-MFCs were firstly connected electrically in parallel and maintained under potentiostatic conditions at 450 mV using the programmable electronic load. The current produced by the cascade was monitored by the voltage drop measured across a wire of known resistance, hence allowing calculation of the produced power.

**Fig. 2:**
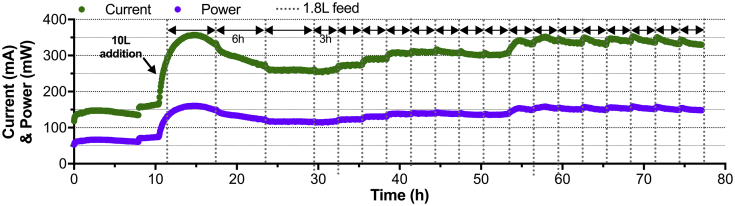
Electrical output of the S-MFC stack comprising a single cascade of four modules. All four modules were electrically connected in parallel and maintained under potentiostatic conditions at 450 mV.

The electronic load was applied 12 h after inoculation. Twelve hours later, once the produced current stabilised around 150 mA, 10 L of freshly collected urine was added to provide fuel and start measuring the current production under regular feeding conditions. At the same time the feeding pulses were set to give the cascade 1.8 L of fuel every 6 h. As soon as the 10 L of fuel were added to the system, the current production increased up to 356 mA in the following 5 h. Although fuel pulses were provided every 6 h, the current dropped to 250 mA during the following 8 h (Fig. 2). Under these running conditions the current production reached a steady-state of 250 mA. At this stage, the feeding pulses were doubled (1.8 L every 3 h) to decrease the HRT of each module to 11.5 h. A new steady-state was reached after 24 h. Under these new feeding conditions, the cascade was producing 337.5 ± 12.5 mA at 450 mV (Fig. 2). This corresponded to a power of 150 mW.

**Fig. 3:**
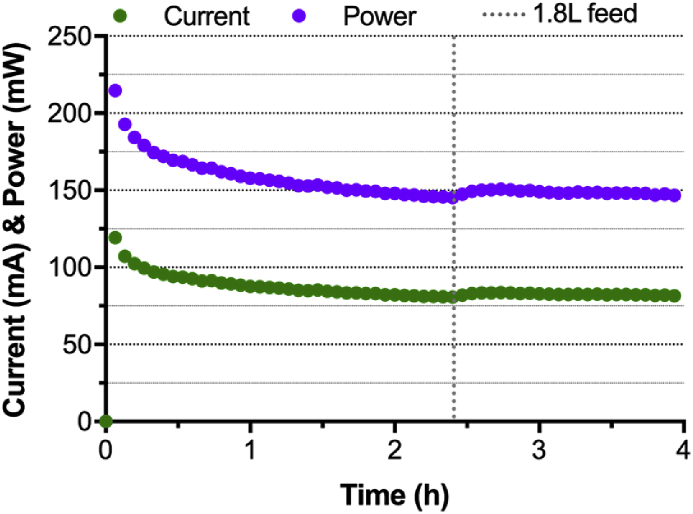
Electrical output of the S-MFC stack comprising a single cascade of four modules. All four modules were electrically connected in series and maintained under potentiostatic conditions at 1800 mV.

These results suggested that each module was producing an average of 84 mA at 450 mV. Therefore, the hypothesis was that under these feeding conditions and if electrically connected in series, the cascade of four modules should be able to produce the 70–75 mA at 1.8 V required to directly power the microcomputer. To verify this hypothesis, all four modules were electrically connected in series. The electronic load was consequently set to 1.8 V. Under this electrical configuration, the cascade displayed a steady-state, over the 4 h observation period, at which it was producing 82.6 mA at 1.8 V (Fig. 3). Compared to the 450 mV potentiostatic conditions, the cascade produced a similar power level of 150 mW suggesting that the performance of the cascade was not affected by the electrical connection in series. Therefore, the hypothesis that the cascade of four modules has the potential to directly power a **GBC** was verified.

### 3.2 Test phase: direct powering of a microcomputer

**Fig. 4:**
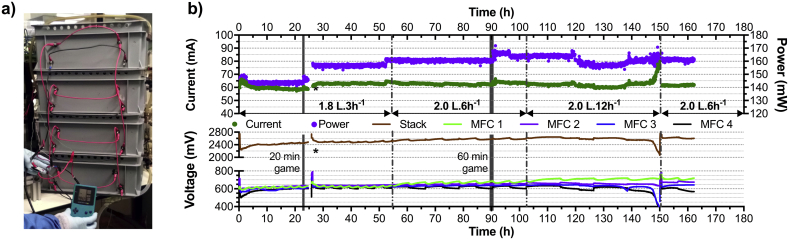
(**a**) Picture of the four modules directly powering a **GBC**. (**b**) Electrical output of the system comprising a single cascade of four S-MFC modules directly connected to a *Game Boy Color*. All four modules were electrically connected in series. Current is the amount drawn by the **GBC**, whilst the voltage is the measured value of each module and of the cascade. The power was calculated using the drawn current and the cascade voltage. * stands for the interruption during which a cascade comprising only the three first modules were connected to the **GBC** (Fig. 5). The grey zones at T = 21 h and T = 87 indicate continuous gameplay.

Based on the results above, the hypothesis was that this cascade could power the **GBC** directly without the use of any power management system or energy buffers such as batteries and/or capacitors (i.e. directly). The next step of this line of work was to directly connect the cascade of four modules, electrically connected in series, to the positive and negative connection of the **GBC**. As for the first phase, the anodes of the first module of the cascade was the negative connection and the cathode of the last module of the cascade was the positive connection. After being left under open circuit conditions for 60 min, the cascade was connected to the **GBC**, which was turned ON (Fig. 4a). It is interesting to note that the **GBC** does not have a sleep mode and will run a demonstration of the game when not in use, hence simulating continuous gameplay. As shown in Fig. 4b, the 20 min and 60 min of continuous gameplay (*Tetris*) did not affect the energy consumption of the **GBC**. Over the first hour, the current drawn by the **GBC** increased from 59 mA to 66 mA whilst the cascade voltage decreased form 2.450 V–2.242 V. Following this, the current drawn decreased to ~59 mA as the voltage increased to ~2.400 V (Fig. 4b). After this initial stabilisation, the current drawn slightly decreased as the voltage increased. Overall, during this first 24 h the power consumption was relatively constant at ~143 mW (Fig. 4b).

**Fig. 5:**
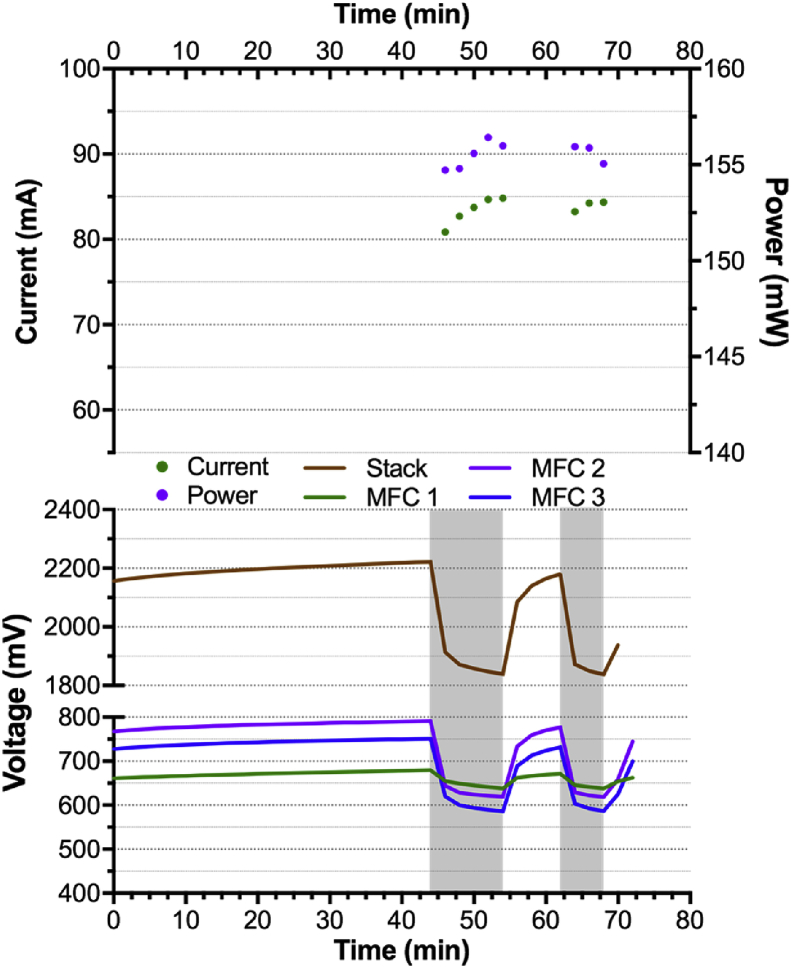
Electrical output of a cascade comprising the three first S-MFC modules electrically connected in series. The grey zones indicate when the **GBC** was ON.

As shown in Fig. 4b, during the first 24 h, the cascade of 4 modules displayed nearly open circuit voltage levels: 2.474 ± 0.010 V (n = 30, from 23.6 h to 24.6 h) compared to the initial 2.740 ± 0.020 V open circuit voltage (n = 6, from 0.16 h to 0.66 h). This implies that the energy drawn by **GBC** from the cascade was much lower than what the cascade could potentially produce, and that a stack of three modules would suffice. Therefore, the cascade was electrically reconfigured as a cascade of 3 modules connected in series (* in Fig. 4b). After 60 min in open circuit conditions, the cascade was directly connected to the **GBC**. Results indicate that the cascade was producing proportionately more power (~52 mW per module) at an increased current of 85 mA. However, after approx. 10 min the voltage dropped below 1.832 V resulting in the **GBC** shutting down (Fig. 5). The system went back to open circuit during the next 10 min prior to the **GBC** being switched ON again. This time, the cascade voltage dropped below the threshold voltage of 1.832 V after 6 min, resulting in the **GBC** switching OFF. This difference in powering time is thought to be due to the capacitance of this type of design: since the cascade did not stay in open circuit for as long as the first time, the apparent capacitance of the cascade did not had sufficient time to build-up as much charge [28]. Therefore, the three-module stack was switched back to four modules connected in electrically in series and connected to the **GBC** to investigate the stability of the produced power over a longer period of time (Fig. 4b).

Due to fuel availability, the stability investigation was carried out for another 24  h at a low feeding rate of 1.8L every 3 h. Results confirmed that the cascade was more than sufficient to power the **GBC** (Fig. 4b). As it was necessary to have four modules connected in series to stay above the threshold voltage of 1.832 V, increasing the system efficiency implied reducing the quantity of fuel supplied to the system. Therefore, the feeding pulses were changed from 1.8L every 3 h to 2.0 L every 6 h. Because of the longer HRT between pulses, the pulsated volume was slightly increased to account for the evaporation losses. Under this new feeding regime, the HRT increased from 11.5 h to 46 h–20.66 h and 82.83 h, for a single module and the cascade respectively. Results show that under this new feeding regime the cascade constantly powered the **GBC** for 48 h (Fig. 4b). Although less fuel was provided to the system, the cascade potential continued increasing from 2.5 V to 2.6 V indicating that the cascade was still able to provide much more energy than what was required by the **GBC**. Following this line of thought, the feeding was reduced to 2.0 L every 12 h, with the subsequent HRT of 41.66 h and 165.66 h, for a single module and the cascade, respectively. Under this feeding regime, the electrical output of the cascade was relatively stable over the first 48 h of the run with an average of 61.6 ± 1.6 mA at a voltage of 2.612 ± 0.023 V (~161 mW). These output levels are comparable to the previous feeding regime (2L.6 h^−1^), that had an average current and voltage of 63.0 ± 0.7 mA and 2.570 ± 0.021 mV (~162 mW). These results show that as a cascade of four modules electrically connected in series, the voltage output was stable whilst being independent from the feeding regime. Conversely, at the module level the voltage levels demonstrated an increasing variability with longer HRT (Fig. 4b). Conversely, the average voltage of the modules was 626 ± 12 mV, 644 ± 23 mV and 653 ± 43 mV for the 1.8L.3 h^−1^, 2L.6 h^−1^ and 2L.12 h^−1^ feeding regime, respectively (n = 900: data points of the last 30 h of each condition).

**Fig. 6:**
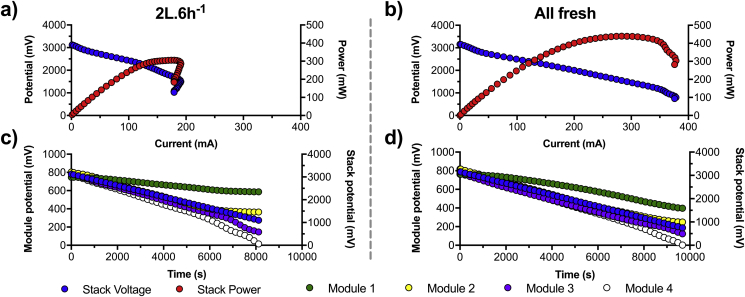
Power and polarisation curves of the 4-modules cascade depending on the feeding conditions: either under a feeding regime of 2L.6 h^−1^ (cascade HRT of 82.83 h; **a** and **c**) or completely filled with fresh fuel (**b** and **d**). (**a**) and (**b**) show the voltage and power curves of the cascades. (**c**) and (**d**) show the voltage of each individual module during the polarisation experiment.

The measured voltage variations can be explained by the fact that the longer the fuel stayed in a module, the more its physico-chemical characteristics changed (e.g. COD/NH_4_^+^ decrease, EC increase). Hence, the downstream modules are fed by increasingly different fuel leading each module to develop slightly different microenvironments, which result in different electrical outputs. This is the main reason why the MFCs of a same cascade that are electrically connected in series are prone to voltage reversal [[11], [12], [13]]. As shown by the results, when the feeding regime was less than 2L.6 h^−1^ the cascade was not producing a stable power output and this resulted in the **GBC** not being powered. Two polarisation experiments of the cascade were performed to evaluate the impact of the feeding regime on the electrical performance of the employed cascade. The polarisation was carried out on the cascade of four modules in a two-electrodes configuration. The first polarisation was performed on the cascade when under a 2L.6 h^−1^ condition whilst the second polarisation run was performed after replacing all electrolyte by fresh urine (Fig. 6).

The results indicate that under 2L.6 h^−1^ conditions the cascade suffered from instability at 1551 mV under a current of 190 mA (Fig. 6a). As seen in Fig. 6c, it is at this stack voltage (i.e. 1551 mV; Blue line, right axis scale) that the modules 3 and 4 began to show mass transfer losses (t = 6320 s; Fig. 6c). Conversely, the voltage curves of modules 1 and 2 indicated that the majority of the losses were ohmic. Hence, the performance drop of the cascade was driven by the last two modules of the cascade and especially the last one that had the most important losses. The polarisation run was stopped when the cascade voltage was 1025 mV because the last module suffered cell reversal (−24 mV). Under 2L.6 h^−1^ conditions the cascade reached a maximum power of 305 mW at 1751 mV and 174 mA. The next day, the cascade electrolyte was totally replaced by freshly collected urine, left 1 h under OCV conditions and then went through a polarisation experiment under a two-electrodes configuration. Under these conditions the OCV of the cascade reached a similar level as the previous run (3150 mV). Compared to the 2L.6 h^−1^ conditions, when replenished with freshly collected urine, the modules displayed divergent voltages much later, at around 950 mV (Fig. 6b). However, only the last module in the cascade showed mass transfer losses and finished the polarisation experiment at 2.2 mV (Fig. 6d). Under these conditions, the cascade produced 439 mW maximum power at 1551 mV and 283 mA. These results indicate that in this study (1) the voltage reversal was mainly due to the feedstock quality difference between the four modules and (2) cell reversal was only measured under high current, higher than was required to continuously power the **GBC**.

## 4 Conclusions

To be used as an energy source Microbial fuel cells are often seen as too unstable and incapable of producing directly exploitable energy levels. Employing power management systems has been a solution exploited so far to remediate the intrinsic current instability and low-power density of microbial fuel cells. Hence, all reports that have demonstrably powered applications with MFCs used power management circuitry. The results presented here demonstrate for the first time that a microcomputer can directly and continuously be powered by MFCs without any energy management apparatus. The 4-module S-MFC cascade continuously produced ≈2.55 V and ≈61 mA, a total of 130 mW, which is above the minimum requirements of the microcomputer and its screen (GBC). However, results showed that producing enough power is not sufficient to directly power an application. Indeed, although the 3-module cascade produced sufficient power to cover the GBC requirements (≈150 mW; ≈82 mA), the voltage was below the threshold level at which the GBC is programmed to switch OFF (1.832 V). This showed that the system had to be calibrated to meet the application-specific needs, which in this case was a voltage higher than 1.832 V. Due to its modular nature, the MFC system can be adapted to any scale of use and yet be energy-positive, which is a feature not always found in other biotechnologies (e.g. biogas). Although the results demonstrated that a MFC system can directly and continuously power a microcomputer, there is room for improvement, especially on scaling the system to the end application. A key aspect that would require attention, especially in terms of employing MFCs as a power source, is to explore the robustness of such system over a long period of time and investigate the stability of the series electrical connection.

## Declaration of interests

The authors declare that they have no known competing financial interests or personal relationships that could have appeared to influence the work reported in this paper.
